# Shall we play together? Game-based learning for engagement and classroom climate in Spanish socially deprived communities

**DOI:** 10.3389/fpsyg.2023.1163441

**Published:** 2023-06-02

**Authors:** José M. Rodríguez-Ferrer, Ana Manzano-León, Carolina Fernández-Jiménez, Antonio Luque de la Rosa, Juan M. Fernández-Campoy, José M. Aguilar-Parra

**Affiliations:** ^1^Department of Developmental and Educational Psychology, Universidad de Jaén, Jaén, Spain; ^2^Health Research Centre, Department of Developmental and Educational Psychology, Universidad de Almería (UAL), Almería, Spain; ^3^Department of Psychology, Universidad de Granada, Granada, Spain; ^4^Department of Education, Universidad de Almería, Almería, Spain

**Keywords:** game-based learning, games, engagement, classroom climate, high school, teenagers

## Abstract

**Purpose:**

The purpose of this study is to analyze the effects of a game-based learning (GBL) program on the classroom climate and engagement of high schools in socially deprived communities in Spain.

**Methods:**

The study included 277 students from two secondary schools located in Southern Spain, situated in Zones in Need of Social Transformation. Sampling was non-probabilistic and accidental, based on the accessibility of the school and the willingness of the management and teaching staff to participate in the GBL program. The study employed a control group and two experimental groups (cooperative games group only and cooperative and competitive games group) to compare pre-test and post-test data in both groups. The Brief Class Climate Scale and Engagement Inventory, validated in academic literature, were used as assessment instruments.

**Results:**

The study used a series of ANOVA tests to compare the experimental groups with the control group. The results indicated statistically significant changes in all study variables. In all cases, the experimental groups demonstrated greater benefits than the control group.

**Discussion and conclusion:**

The study findings reveal that games can provide significant benefits to students, regardless of whether they are cooperative or competitive. The study provides evidence of the benefits of GBL in high schools located in socially deprived communities in Spain.

## 1. Introduction

### 1.1. Classroom climate in socially deprived communities

Classroom climate refers to the perceptions and opinions of students and teachers about the educational environment within the classroom (Villanueva, 2020). During adolescence, classroom climate is especially critical as it is a crucial stage in emotional development and where students spend most of their time socializing in school (Wang and Eccles, 2012). A positive classroom climate is characterized by respectful and emotionally supportive relationships between teachers and students. In contrast, a hostile classroom climate implies a lack of emotional connections between teachers and students, which can encourage disrespect, insults, and even aggression (Cohen, 2021), leading to academic demotivation and emotional disconnection with education (Sakiz, 2012). Extensive research has been conducted on the relationship between teachers and students, concluding that teachers should be emotionally competent and offer sympathetic treatment to students to help them feel respected in the classroom (Brackett, 2019; García-Moya et al., 2020). They should also create motivating learning situations that can promote positive feelings toward learning (LeBlanc, 2022). In the meta-analysis study by Wang et al. (2020), it is evident that classroom climate has a positive relationship with social competencies, motivation, engagement, and academic performance while damaging relationships with socioemotional distress and antisocial behaviors.

In Spain, Decree-Law 7/2013, of 30 April 2013, on extraordinary and urgent measures for the fight against poverty and social exclusion (BOJA no. 85 of 03/05/2013) defines Zones in Need of Social Transformation (ZNTS) as specific and physically delimited urban spaces where the population experiences severe poverty and social marginalization. Socioeconomic difficulties, such as deterioration or deficits of infrastructures and public services, high unemployment rates, hygienic-sanitary deficiencies, and low parental education, can manifest in these areas, leading to an inability to accompany students to school. This can also result in socio-educational difficulties related to social exclusion, high absenteeism rates, and school failure (Fernández-García et al., 2019).

Children and teenagers socialized in ZNTS, socially deprived communities, may exhibit antisocial and violent behavior patterns that directly affect the classroom climate and negatively manage classroom conflicts (Narváez Burbano et al., 2020). Therefore, their education requires a set of general and specific measures and resources to facilitate flexible groupings, preventive programs, open and flexible organization of spaces and times, an adaptation of didactic programs, and educational reinforcement to compensate for possible family, economic, and sociocultural deficiencies and reinforce the learning of basic skills (Ruiz-Román et al., 2019). Creating and strengthening peer relationships have been critical in generating a positive classroom climate (Urdan and Schoenfelder, 2006; Okada, 2021). To achieve this support, teachers should create learning situations that allow peer relationships and cooperation (Cecchini Estrada et al., 2019).

### 1.2. Engagement and academic motivation

Engagement in education refers to the degree to which students are immersed in classroom activities (Clynes et al., 2020). The more engaged the students are, the more focused and participative they are likely to be in the task (Bergdahl et al., 2020; Xie et al., 2020). Engagement is influenced by various factors, including teaching style, sociocultural context, and motivation toward the task (Kahu et al., 2020). Behaviors related to student engagement are dynamic and interconnected with the environment. In socially deprived communities, students often exhibit lower academic motivation and low expectations regarding their abilities, leading to academic demotivation, absenteeism, and school failure (Ricard and Pelletier, 2016; Artuch-Garde et al., 2017). Engagement is associated with positive emotions that enable student participation (Shelton-Strong and Mynard, 2021). High engagement leads to positive changes in behavior and represents a proactive attitude toward learning, leading to reduced school dropouts (Marôco et al., 2020; Abreu Alves et al., 2022).

Engagement comprises three interrelated dimensions: emotion or affect, behavioral aspect, and cognitive aspect (Christenson et al., 2012). Emotion refers to the positive emotions experienced and the absence of negative emotions in the environment, which encourages the student to continue in that situation (Skinner et al., 2008). The behavioral aspect refers to all the energy mobilized to satisfy expectations related or unrelated to learning, which is associated with the social and cultural context (Medrano et al., 2015). Finally, the cognitive aspect refers to the cognitive strategies that the learner performs to achieve the set goals (Galikyan and Admiraal, 2019). Engagement is closely related to motivation, and intrinsic motivation is a prerequisite for engagement to occur (Delaney and Royal, 2017). Engagement protects against school dropouts (Álvarez-Pérez et al., 2021) and is positively associated with school performance (Estévez et al., 2021) and socioemotional wellbeing (Wang et al., 2019). Intrinsic motivation and actual engagement positively correlate and influence learning outcomes (Saeed and Zyngier, 2012). Teachers should use educational strategies that promote motivation and meaningful learning to enhance engagement and prevent student disengagement (Balwant, 2018; Lira Munizaga and Pérez-Salas, 2022).

Students in ZNTS are at a higher risk of failing to complete their studies successfully (Rydell, 2010) and exhibit lower motivation and academic achievement (Leggett and Harrington, 2021). Therefore, measures and resources should be put in place to support these students, such as flexible groupings, preventive programs, an adaptation of didactic programs, and educational reinforcement to compensate for possible family, economic, and sociocultural deficiencies and reinforce the learning of basic skills (Ruiz-Román et al., 2019). Engagement can help students overcome these challenges and succeed in their academic pursuits.

### 1.3. Game-based learning

Games have the potential to facilitate cognitive and behavioral changes and can be used as learning resources (Buelow et al., 2015; Krath et al., 2021). Playful activities are associated with the need for expression and the search for gratifying emotions, and this dimension of human development is known as the playful universe, or homo ludens (Bayeck, 2020). Games are cultural phenomena that can provide rewarding learning experiences. The educational field has shown interest in exploring games and playful strategies as innovative learning spaces (Wu et al., 2012; Manzano-León et al., 2021a,b) to promote esthetic experiences of enjoyment and generate differences from traditional didactic materials that only focus on content (Barría, 2022).

Game-based learning (GBL) is the systematic use of analog or digital games to work on specific contents or skills previously established by the teacher (Cornellà et al., 2020). GBL can be used in education through two channels: a rational and analytical channel that highlights its formal structure formed by rules, mechanics, dynamics, and procedures, and an emotional and experiential channel that highlights those elements that motivate players, such as fun, competition, cooperation, or challenges (Olejniczak et al., 2020).

Although GBL has shown many possibilities and benefits, potential limitations have also been noted, such as the negative perception of play by families and even teachers as a distraction to learning (Kirstavridou et al., 2020) and the discomfort and stress that game competition can cause for some students (Jääska and Aaltonen, 2022). Students may also prefer traditional learning methods due to the difficulty and demand of gamified lessons (Scepanovic et al., 2015).

Learning is linked to play through motivation as playing is enjoyable for humans and can serve as an engine for learning different contents, values, and competencies. Playful learning is theorized to foster greater motivation and fun while working on concepts, skills, and behaviors (Fulya Eyupoglu and Nietfeld, 2019). GBL aims to achieve greater motivation and fun while working on these same concepts, skills, and behaviors. Recent research has highlighted the simplicity of GBL's mechanics and game dynamics, as well as its affordability and accessibility, which favor its use in both formal education and informal learning environments (Wonica, 2017).

At a conceptual level, GBL can be a refreshing approach to learning in secondary education, fostering knowledge construction while encouraging creativity and imagination. Play involves learning as participants engage with play and learn to interact with play (Steinkuehler et al., 2012). GBL can also support constructivist learning, where learning is embedded in participation, engagement, and interaction with and around games (Gee, 2005). Learning occurs not only through knowledge acquisition or behavioral change but also in the various practices and interactions that players engage in within the play experience (Ke et al., 2016).

### 1.4. Research objectives

This study focuses on the effects of a GBL program on the classroom climate and engagement of high school students in socially deprived communities. It is a longitudinal quasi-experimental study (pre-post-test) conducted among Spanish high school students. The program designed and evaluated a board game initiative during a 12-week tutoring period to promote positive coexistence. While there is evidence of a positive relationship between games and classroom climate (Huizenga et al., 2019; Coleman and Money, 2020), gamification on engagement and flow (Manzano-León et al., 2023) has been studied, and the application of GBL to the study of classroom climate with socially deprived communities is underexplored. This study aims to address the following research questions:

- Does the GBL influence the student's perception of the classroom climate?
- Does the implementation of GBL have any impact on student engagement?
- Does selecting games [cooperative only or mixed (cooperative and competitive)] influence school climate and engagement?

## 2. Materials and methods

### 2.1. Participants

The selected sample for this study consisted of high school students from nine classes located in the southern region of Spain within ZNTS. Randomization was used to determine which classes would participate in the control group and which classes would be assigned to the experimental group. The sample size and distribution are shown in Table 1.

**Table 1:** Study participants.

	**Sex**		**Age**		**Total *N***
	**Men**	**Female**	**M**	**DT**	
Control	57	54	13.49	1.48	111
Experimental 1 (cooperative games)	38	34	14.66	1.46	72
Experimental 2 (cooperative and competitive games)	49	45	14.79	1.47	94
Total	144	133	13.55	1.47	277

The sample was non-probabilistic and selected based on the accessibility of the schools and the willingness of the management and teaching staff to participate in the GBL initiative. To be eligible for either the experimental or control group, students had to meet the following criteria: students should (a) be enrolled in ZNTS; (b) be between the ages of 13 and 16 years; and (c) have attended at least 60% of the tutoring classes during the period being evaluated.

Before data collection, the students were fully informed about the nature of the research and were assured of anonymity. The GBL program was integrated into the tutoring curriculum. This study adhered to the recommendations of the American Psychological Association and the Declaration of Helsinki, and ethical approval was obtained from the Research Ethics Committee of the University of Almería (ref. 01/2021).

### 2.2. Instruments

The Brief Classroom Climate Scale (Bisquerra and López-Gonzalez, 2013) is a questionnaire consisting of 11 items, which are divided into two dimensions (Group cohesion and Group leadership) and five subdimensions (satisfaction and involvement, peer cohesion, teacher–student relationship, order and organization, and task orientation). The items are classified on a four-point Likert scale, which presents four response options: never, sometimes, frequently, and always. The total scale has a high internal consistency, with a Cronbach's alpha value of 0.83.

The Engagement Inventory (Wang et al., 2014) is a questionnaire that has been validated for use with Spanish-speaking populations through a confirmatory factor analysis (Manzano-León et al., 2021a,b). The questionnaire assesses students' engagement and consists of items that measure behavioral, cognitive, and emotional engagement. The internal consistency of the questionnaire is high, with Cronbach's alpha values exceeding 0.80.

### 2.3. Procedure

To address the research questions, a quasi-experimental longitudinal design with pre-post-evaluation and a control group was conducted. Before the intervention, a preliminary assessment was carried out to ensure equivalence between the groups on the variables under study. The experimental group received 12 sessions of a GBL workshop in the tutoring subject of the 1st and 2nd years of high school, while the control group watched videos related to tutoring, completed reading assignments, and did homework on other subjects during tutoring classes. After the intervention, the same questionnaires were administered during school hours. It was agreed with the participating schools that if the GBL program was beneficial for the students, the control group classes would participate in the following school year.

For the GBL workshop, commercial board games that could effectively enhance classroom climate and student engagement in the tutoring class were selected by researchers who specialized in ludic strategies. The GBL program was designed using competitive and cooperative dynamics in the chosen games. Competitive games have traditionally been used to create an enjoyable experience for players, allowing them to stay interested in the activity for longer (Camacho-Sánchez et al., 2023), which can increase motivation and participation. However, using only competitive dynamics can create a tense and even violent atmosphere (Adachi and Willoughby, 2011). On the other hand, cooperative games can promote relationships between participants and allow for more social interaction, encouraging socialization and greater understanding between people (Creighton and Szymkowiak, 2014).

The selected games had a variety of game mechanics, dynamics, and esthetically appealing features to young adolescent audiences. The games selected were as follows (see Table 2).

**Table 2:** Selection of board games.

**Name (editorial)**	**Game type**	**Components**	**Game objective**
*Batalla de genios* (Lúdilo)	Competitive	Board and dice	Be the first to place the pieces in an orderly sequence, avoiding the wooden obstacles indicated by the dice.
*Camel up* (Más que Oca)	Competitive	Dice, cards, and board	Earn as much money as possible by supporting the Camel they believe will win each Stage and the one that will win and lose the entire race.
*Días de Radio* (Guerra de Mitos)	Cooperative	Cards	Narrate a story in real-time, using ideas from the scripts (letters) provided by their classmates.
*Isla prohibida* (Devir)	Cooperative	Board and cards	Collect the four treasures on the island without any player being cut off or the tide rising high enough. Players must cooperate to collect the treasures, secure the grounds, and reach the helicopter.
*Rhino Hero* (Haba)	Competitive	Cards	Build a tower as high as possible, selecting special effects from your cards, such as having another player draw cards, jumping turns, or placing Rhino Hero (a piece of wood) on top of the tower.
*Sherlock Q* (Guerra de Mitos)	Cooperative	Cards	Solve a mystery from the cards, reviewing important information and solving direct and inferential questions.
*Sí Señor Oscuro* (Asmodee)	Competitive	Cards	Make excuses with the text or images on the cards and incriminate another player
*Speed Cups* (Mercurio)	Competitive	Cutlery, cards, and bell	Be the first to arrange the colored cups as indicated on the cards and ring the bell.
*Story cubes* (Asmodee)	Cooperative	Dice	Tell or write an invented story related to the dice drawings.
*Taco, gato, cabra, queso, pizza* (Ludilo)	Competitive	Cards	Be the first player to run out of cards. When a player discards a card that matches the word he says, he places his hand on the center pile; the last player to place his hand on the center pile gets all the cards in the pile.
*Virus* (Tranjis Games)	Competitive	Cards	Get a healthy body (4 organ cards) in your game space. In the game, you can place organs, put viruses on others, cure viruses, or use cards with special effects.

Source: own elaboration.

To address the third research question, the experimental group was divided into two subgroups: one played only cooperative games, and the other played all the selected games, both cooperative and competitive. The workshop was conducted during one trimester of the tutoring course, with 12 1-h sessions during school hours. The classroom teacher and two principal investigators jointly conducted the workshop. Before starting the program, the teachers received training on GBL, and the games used in the program.

### 2.4. Data analysis

The data processing for this research study utilized R Studio software in version 4.01 with the Tidyverse package. To calculate the direct scores for each factor containing the instruments used in this research, responses from the participants in each group were taken and processed according to the manuals of the instruments.

Before starting the statistical analysis, an ANOVA test was conducted to verify the equivalence of the groups at the start of the investigation, using the pre-test scores. To answer the research questions, another ANOVA test was conducted using the post-test scores of the participants, with *post-hoc* tests conducted after statistically significant differences were determined.

The Bonferroni adjustment method was used for *post-hoc* tests, and ANOVA tests were chosen instead of *t*-tests to avoid type 2 errors. This decision was made due to the sample size potentially causing the accumulation of small differences to be interpreted as statistically significant differences when there may be no meaningful differences.

## 3. Results

The analysis of the results was guided by the research questions posed in this study. Therefore, this section has been structured according to the research questions. Table 3 reports the means and standard deviations of the control and two experimental groups (cooperative and competitive-cooperative).

**Table 3:** Means and standard deviations of the variables studied before and after the intervention.

	**Control**		**Competitive**		**Competitive-cooperative**	
	**M**	**SD**	**M**	**SD**	**M**	**SD**
**Pre engagement inventory**						
Affective motivation	16.67	3.05	17.49	3.48	17.37	3.23
Motivation behavior	16.00	2.82	16.67	2.82	16.18	2.68
Class participation	16.65	2.97	17.34	2.83	17.32	2.92
Cognitive motivation	33.63	6.76	34.53	6.34	33.74	6.04
Disengagement	6.38	2.39	5.89	2.74	6.10	2.30
**Pre-classroom climate**						
Satisfaction climate	6.40	1.46	6.30	1.50	6.19	1.59
Cohesion climate	5.94	1.42	5.74	1.27	6.06	1.55
Full cohesion	12.33	2.48	12.04	2.28	12.26	2.70
Relationship climate	5.82	1.27	5.67	1.05	5.65	1.30
Order climate	5.35	1.20	5.36	1.24	5.19	1.07
Orientation climate	7.41	1.68	7.34	1.29	7.26	1.58
Driving climate	18.59	3.43	18.37	2.73	18.10	3.16
**Post engagement inventory**						
Affective motivation	16.70	2.43	18.87	2.96	19.59	2.35
Motivation behavior	16.47	2.54	17.49	2.39	18.40	2.57
Class participation	17.13	3.01	18.97	3.32	18.69	3.34
Cognitive motivation	34.18	6.79	42.27	7.04	42.76	6.39
Disengagement	5.58	2.15	4.64	1.52	4.43	1.28
**Post-classroom climate**						
Satisfaction climate	5.92	0.96	7.64	1.04	7.61	1.23
Cohesion climate	6.03	1.23	6.94	1.51	7.04	1.35
Full cohesion	11.96	1.63	14.58	2.08	14.65	2.12
Relationship climate	6.08	0.98	7.36	1.24	6.92	1.25
Order climate	5.76	1.01	6.20	1.20	6.07	1.02
Orientation climate	7.45	1.35	8.50	1.59	8.26	1.49
Driving climate	19.29	2.42	22.08	3.02	21.26	2.75

The initial comparison between groups was conducted using the pre-intervention scores, and an ANOVA test was performed for each study variable. The results of the analysis are presented in Table 4. The statistical tests showed no statistically significant differences (*p* < 0.05) between the groups for any of the variables analyzed. Therefore, it can be concluded that the groups started from a statistically equal baseline in the variables analyzed in this research.

**Table 4:** ANOVA tests of variables on scores before intervention.

	***F***	***p***	** $\eta_{p}^{2}$ **
**Pre engagement inventory**			
Affective motivation	2.883	0.091	0.01
Motivation behavior	0.293	0.589	0.001
Class participation	3.181	0.76	0.011
Cognitive motivation	0.129	0.72	< 0.001
Disengagement	0.686	0.408	0.002
**Pre-classroom climate**			
Satisfaction climate	0.617	0.85	0.003
Cohesion climate	0.411	0.522	0.001
Full cohesion	0.036	0.849	< 0.001
Relationship climate	0.732	0.849	< 0.001
Order climate	0.799	0.372	0.003
Orientation climate	0.308	0.579	0.001
Driving climate	0.873	0.351	0.003

The analysis of the first research question regarding the students' perception of classroom climate shows that ANOVA tests performed with the scores obtained after the intervention was completed yielded statistically significant differences (*p* < 0.05) between the control group and the experimental groups in all study variables. Table 3 demonstrates that these differences were in favor of the experimental groups, indicating that the intervention positively modified the classroom climate perception in the experimental groups. Another way to reinforce this statement is to observe the effect sizes found in the statistical analysis, reported by partial eta squared (η_p^2^), which ranged from moderate (0.02–0.09) to strong sizes (>0.09).

The analysis of the first research question regarding the students' perception of classroom climate shows that ANOVA tests performed with the scores obtained after the intervention was completed yielded statistically significant differences (*p* < 0.05) between the control group and the experimental groups in all study variables. Table 3 demonstrates that these differences were in favor of the experimental groups, indicating that the intervention positively modified the classroom climate perception in the experimental groups. Another way to reinforce this statement is to observe the effect sizes found in the statistical analysis, reported by partial eta squared ($\eta_{p}^{2}$), which ranged from moderate (0.02–0.09) to strong sizes (>0.09).

Regarding the second research question, which deals with student engagement, ANOVA tests were also performed with the scores obtained after the intervention, showing statistically significant differences (*p* < 0.05) between the experimental and control groups in the variables studied. *Post-hoc* tests revealed differences in favor of the experimental groups. Effect sizes found varied from moderate to strong, suggesting that the intervention modified student engagement, increasing it in the experimental groups (see Table 5).

**Table 5:** ANOVA and *post-hoc* tests for scores after intervention.

	***F***	***p***	** $\eta_{p}^{2}$ **	***Post-hoc***
**Post-engagement inventory**				
Affective motivation	67.71	^***^	0.197	Control-Exp1^***^/Exp1-Exp2^***^
Motivation behavior	28.08	^***^	0.092	Control-Exp1^***^/Exp1-Exp2 = 0.03
Class participation	12.75	^***^	0.044	Control-Exp1^***^/Control-Exp2^***^
Cognitive motivation	80.09	^***^	0.225	Control-Exp1^***^/Control-Exp2^***^
Disengagement	5.365	^***^	0.069	Control-Exp1^**^/Control-Exp2^***^
**Post-classroom climate**				
Satisfaction climate	112.47	^***^	0.29	Control-Exp1^***^/Control-Exp2^***^
Cohesion climate	26.23	^***^	0.087	Control-Exp1^***^/Control-Exp2^***^
Full cohesion	90.18	^***^	0.246	Control-Exp1^***^/Control-Exp2^***^
Relationship climate	24.83	^***^	0.083	Control-Exp1^***^/Control-Exp2^***^
Order climate	4.32	0.03	0.015	Control-Exp1^*^
Orientation climate	14.19	^***^	0.049	Control-Exp1^***^/Control-Exp2^***^
Driving climate	24.36	^***^	0.081	Control-Exp1^***^/Control-Exp2^***^

* *p* < 0.5;

** *p* < 0.01;

*** *p* < 0.001;

Control, control group; Exp1, group cooperative games; Exp2, cooperative and competitive group games.

Finally, the third research question aimed to explore whether the mechanics used in the experimental groups, one with only cooperative games and the other mixing competitive and cooperative games, had any effect on the perception of classroom climate and student engagement. To answer this question, *post-hoc* tests were conducted and are shown in Table 5. No statistically significant differences were found when comparing the experimental groups. However, differences were found between the control group and each of the experimental groups. Therefore, it can be deduced that the mechanics used in the groups did not have a statistically significant influence on the variables studied.

## 4. Discussion and conclusion

The ZNTS faces significant economic and social challenges that require a comprehensive approach to promote sustainable and equitable development (Chapman and Ainscow, 2019; Vela-Jiménez et al., 2022). Providing quality educational opportunities that give access to students to improve their living conditions and have a promising professional future is one of these challenges. Barriers to delivering such education include limited access to education, inadequate infrastructure, and gender equity issues (Cárdenas-Rodríguez et al., 2018). Additionally, Abuya et al. (2013) identify the general lack of motivation toward school as another obstacle. Therefore, finding educational methodologies and strategies to promote student engagement and motivation in compulsory education is a leading topic in educational research (Rumberger and Rotermund, 2012; Keyes, 2019). Among these methodologies, game-based learning and gamification are increasingly prominent (Abdul Jabbar and Felicia, 2015; Pratama, 2020; Jayawardena et al., 2022). This research aims to integrate a GBL experience to evaluate its impact on the engagement and classroom climate of Spanish high school students in ZNTS.

In response to the first research question (does GBL influence students' perception of the classroom climate?), this study provides new insights into GBL as an effective strategy for improving the classroom climate in ZNTS. The results demonstrate that students who played board games during tutorials experienced statistically significant improvements in all the variables studied. However, it should be noted that some effect sizes were small, which may limit their impact on individuals, such as in the classroom climate and climate orientation within the climate scale, and class participation in the engagement inventory. Despite these limitations, students who played board games during tutorials exhibited improvements in the study variables compared to those who attended regular tutoring sessions. These findings align with previous research reporting the benefits of GBL in the classroom (Pinedo et al., 2022). Therefore, board games can be a valuable resource for students, improving the classroom climate through game mechanics and dynamics that favor cooperation, communication, and conflict resolution, leading to better group dynamics and a more positive and welcoming classroom environment (Smith and Golding, 2018; Bauserman et al., 2021).

After answering the second research question (Does the implementation of GBL impact student engagement?), this study examined GBL as an influential variable in student engagement in ZNTS. The research compared the engagement and disengagement of the three groups (control, experimental group of cooperative board games, and experimental group of competitive and cooperative board games). The results indicate statistically significant improvements in all the variables studied, with the motivation variables of the engagement inventory and the total cohesion of the classroom climate experiencing significant changes. The increase in motivation coincides with the affirmation of board games in the classroom as a motivating activity, as indicated by previous studies (Acquah and Katz, 2020; Teixeira et al., 2022).

Revised: With regard to the comparison between the groups that played competitive games and a mixture of competitive and cooperative games, the study found no statistically significant differences in any of the study variables. This aspect of the study is significant because it explores how the type of game affects social behavior in the classroom, which has yet to be previously investigated.

The focus of this study is to inform the scientific and educational community about the potential of GBL with both cooperative and competitive board games for improving classroom climate and engagement, particularly in ZNTS. However, the main limitation of this research is the sample size of secondary school students, which is limited to a single Spanish city with ZNTS. To further validate the findings, future research should replicate this investigation in other disadvantaged contexts. Additionally, studying the long-term effect (follow-up test) of the use of GBL on the variables studied, as well as the influence of GBL on other variables of interest such as academic performance, emotional intelligence, and school absenteeism, should be considered in future studies. Overall, it can be concluded that GBL can be an innovative and effective educational methodology to promote a positive classroom climate. Board games allow students to interact with each other in a playful environment, which encourages cooperation, communication, and conflict resolution among students, leading to better group dynamics and a more positive and welcoming classroom environment.

## Data availability statement

The original contributions presented in the study are included in the article/supplementary material, further inquiries can be directed to the corresponding author.

## Ethics statement

The studies involving human participants were reviewed and approved by the Research Ethics Committee of the University of Almería (ref. 01/2021). Written informed consent to participate in this study was provided by the participants' legal guardian/next of kin.

## Author contributions

JR-F and AM-L contributed to the conception and design of the study. JR-F organized the database and performed the statistical analysis. AM-L wrote the first draft of the manuscript. CF-J, AL, JF-C, and JA-P revised sections of the manuscript. All authors contributed to the manuscript revision, read, and approved the submitted version.
